# Developing Hydration Maps of Polymer Latex Film Formation Using Terahertz Time-Domain Spectroscopy

**DOI:** 10.1021/acs.langmuir.4c03103

**Published:** 2024-11-13

**Authors:** Gonçalo Costa, Emily M. Brogden, Jacob J. Young, Huiliang Ou, Arturo I. Hernandez-Serrano, Rayko I. Stantchev, Stefan A. F. Bon, Emma Pickwell-MacPherson

**Affiliations:** † Department of Physics, University of Warwick, Gibbet Hill Road, Coventry CV4 7AL, U.K.; ‡ Department of Chemistry, University of Warwick, Gibbet Hill Road, Coventry CV4 7AL, U.K.; § Department of Physics, National Sun Yat-sen University, Kaohsiung 80424, Taiwan

## Abstract

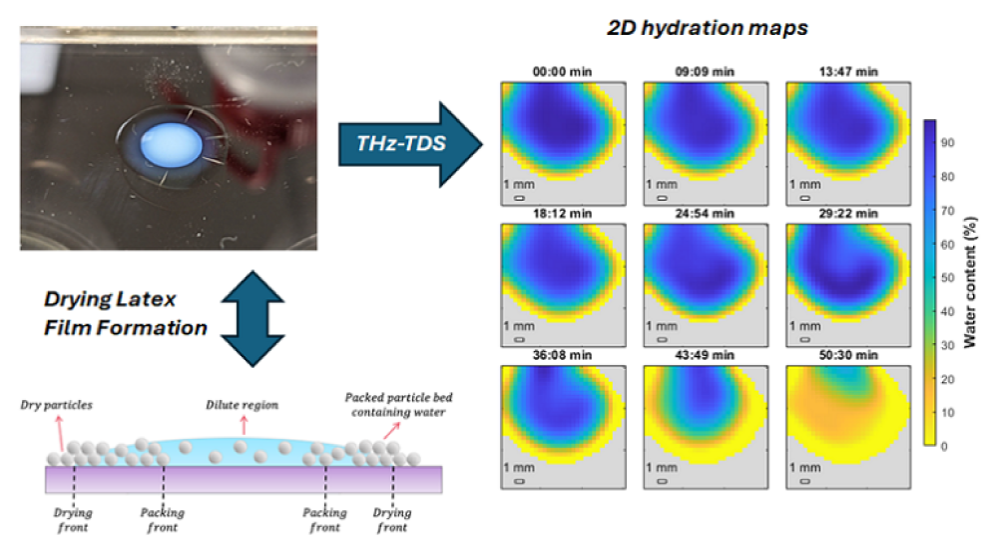

The dynamics of the drying process of polymer latexes after casting as a wet film onto a substrate are important to track as they influence the physical and mechanical properties and performance of the dried polymer films. Current methods used to follow this drying process include gravimetric analysis, coupled with advanced techniques like GARField-NMR or optical coherence tomography. The latter two methods provide height and spatial information in the *z*-direction, normal to the substrate, and occasionally in the xz- or yz-planes. Terahertz time-domain spectroscopy (THz-TDS) is a welcome addition as it provides both the structural and spectroscopic information in the parallel xy-plane, filling the geometric gap. Herein, we utilize THz-TDS to study the drying and film formation process of various polymer latexes with a broad range of glass transition temperatures. We showcase the applicability of this technique in obtaining 2D parallel hydration maps of the drying dispersions, in the form of droplets, using latex-dependent calibration lines. Our findings display known phenomena arising from the drying of the colloidal dispersions.

## Introduction

**Figure 1:**
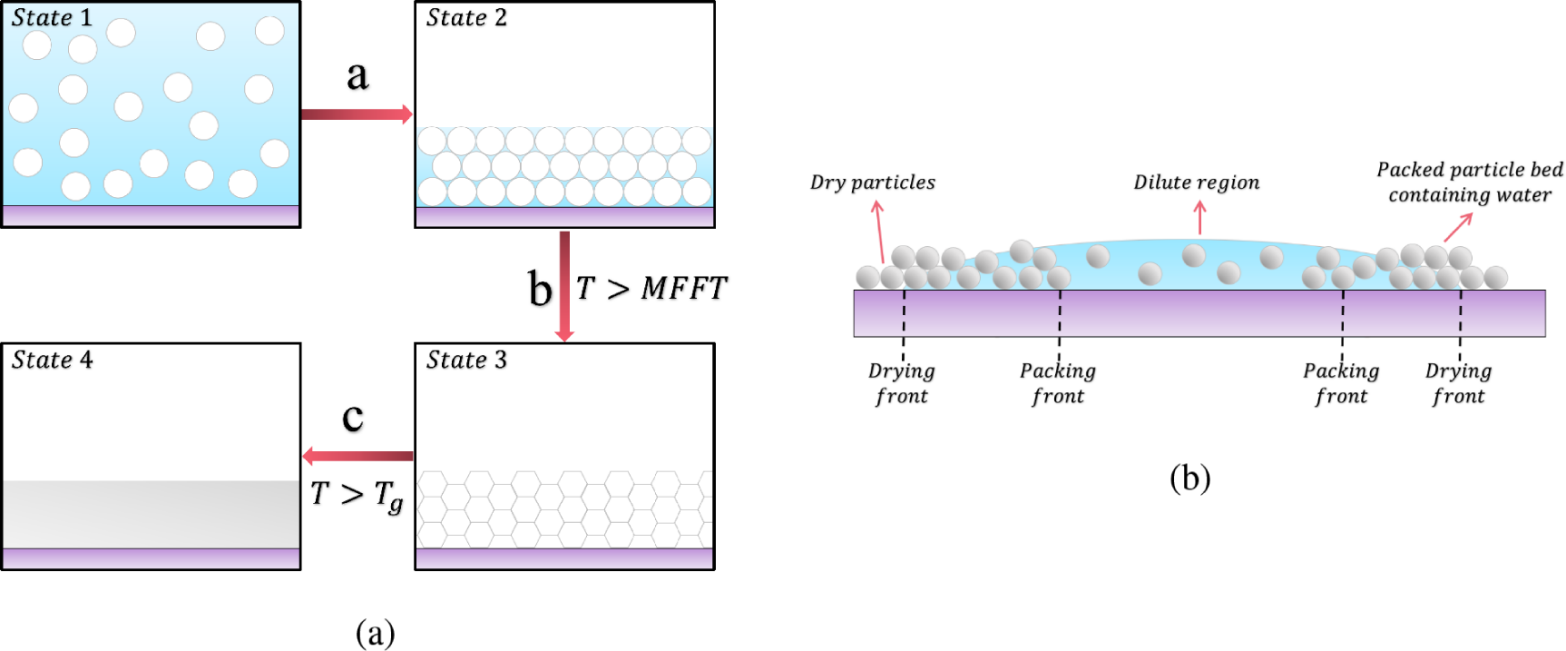
(a) Schematic diagram of the idealized drying stages of a wet polymer latex film without *xz*- and *yz*-boundaries (infinite film of specific thickness), with film formation occuring above the minimum film formation temperature (MFFT) so that particles can deform and polymer chains can interdiffuse. (b) A schematic diagram of the drying and packing fronts of a latex droplet or film using particles of high glass transition temperature that do not deform.

Polymer films produced through solvent evaporation during the drying of a dispersion of polymer particles are used in a wide range of industries and include products such as sealants, protective coatings, adhesives, inks and paints. The drying conditions of a casted wet latex film can significantly influence the mechanical properties of the dried polymer film. An understanding of this film formation process is therefore essential for the success of many commercial products,^1−6^ and many studies have been conducted.^7−13^Figure 1 depicts two scenarios of the drying process of water-based dispersions of polymer colloids. In the first case a wet film (commonly 5 μm–2 mm in thickness after casting) where the boundaries in the *xz*- and *yz*-planes are ignored (an infinite film of specific thickness), is considered. Figure 1a shows how a polymer latex (particle diameter typically 50–800 nm with a glass transition temperature, *T*_*g*_, below the film formation temperature) becomes a continuous and homogeneous polymer film. This film formation process occurs in four stages. First the dispersion concentrates and the particles pack together as the water evaporates (state 1 to state 2). Then the particles deform driven by a capillary underpressure as the remaining water is lost from the interstitial spaces between the latex particles (state 2 to state 3). The final stage (state 3 to state 4) known as polymer–polymer interdiffusion occurs over a long time period, ranging from hours to weeks, and depends on the polymer chemical composition and chain architecture. Here, phase boundaries of the previously existing particles disappear to eventually produce a continuous polymer film. The second scenario illustrates one where a uniform wet film height is not possible because of the edges of a drying film or droplet. This is illustrated in Figure 1b with latex particles of a *T*_*g*_ considerably higher than the drying temperature so that the original spherical shape of the particles remains intact throughout the entire drying process. Drying fronts that appear are attributed to a reduced height at the edge of a film, where the capillary pressure keeps the water at the film surface. Evaporation of water from this contact line is faster triggering an outward Deegan flow, resulting in an accumulation of particles by capillary action from the center of the drying film to the contact line. This nonhomogeneous lateral drying is also known as the “coffee-ring” effect. In most cases, the center of a droplet or film is the last to dry.

One of the most common and practically straightforward methods to study the drying and film formation of water-based polymer dispersions is gravimetry. This technique monitors the weight of the drying latex using a digital balance to calculate the water content and rate of evaporation, over time.^14−17^ Gravimetric analysis does not probe the sample spatially and can only provide the average overall water content and evaporation rate. It can be combined with more complex techniques that provide additional information, such as height or lateral cross-sectional data. For example, Optical Coherence Tomography (OCT) uses a near-IR light source to obtain a sample’s cross-sectional image with a penetration depth of up to 1–2 mm.^18^ Drying rates, cracking phenomena, and speckle images of the sample’s internal structure can be obtained using this technique in combination with gravimetry.^19^ Alternatively, Gradient At Right Angles to the Field Nuclear Magnetic Resonance (GARField-NMR), generates intensity profiles which are proportional to the density of mobile ^1^H atoms, enabling real-time acquisition of the water distribution in a drying film.^9,16^ These techniques, while useful, give limited information on the water content of a drying sample in the *xy*-plane, parallel to the substrate the film is drying on. We show herein how Terahertz Time-Domain Spectroscopy (THz-TDS) can be used as a complementary technique to obtain additional information in the *xy*-plane over relatively large samples areas (1–3 cm), hence 2D_*xy*_.

Terahertz (THz) radiation lies in the wavelength range between 30 μm and 3 mm, between the microwave and infrared regions of the electromagnetic spectrum. Its non-ionizing properties deem it ideal for analyzing a broad range of samples from plastics^20^ to paintings,^21^ using techniques like THz-TDS. These methods allow for the acquisition of spectroscopic images with consistent spatial resolution. The THz frequency range coincides with the absorption region of the intermolecular interactions within hydrogen-bonded networks, such as those in water and proteins. This characteristic has been utilized in a wide range of studies, including protein spectroscopy,^22^*in vivo* skin analysis^23^ and imaging,^24^ as well as studying the moisture content of paper.^25^

THz-TDS has been previously used to study thin films, showing promise due to its nondestructive and contactless nature. This technology has been used to measure the height of drying paint films,^26−28^ the layers within a multilayer structure^29,30^ and defects between layers.^31^ A study by van Mechelen provides insight into the stratification of waterborne and solvent-borne colloidal dispersions during drying by measuring the thickness of paint layers (notably greater than 10 μm thick) thus observing the wet and dry layers as a function of drying time.^32^ While this study provided useful insights, it did not map the drying process over a large section of the sample and lacks quantitative data on water content. Herein lies the great advantage of using THz radiation to monitor the drying process: it can provide direct information on the hydration dynamics of a drying polymer dispersion as it forms a film.

We take advantage of THz sensitivity to water and its nondestructive nature to study the drying process and film formation of droplets of water-based polymer colloids. The polymer latexes used were made by emulsion polymerization. We show that THz-TDS has the ability to image a range of polymer dispersions with different *T*_*g*_s, and discuss in depth the advantages and limitations of the technique.

## Experimental Section

### Instruments

The average hydrodynamic particle diameter, *d*_*z*_, and dispersity index, *PDI*, of the polymer latexes were recorded using an Anton Paar Litesizer 500 (0.3–2,000 nm). Differential scanning calorimetry (DSC) measurements were carried out on a TA Instruments DSC2500 for Latex 1 and 3 and Metler Toledo STAR^*e*^ instrument for Latex 2. THz-TDS was performed using two different Menlo Systems GmbH time-domain spectrometers (TERA K15 and Terasmart) to ensure reproducible results between systems. The THz-TDS systems were staged in a reflection setup with the emitter and detector set at an incident angle of 30^◦^ to the quartz imaging window on the first setup, and of 8.8^◦^ for the second. A Metler Toledo Analytical Balance ME204 was used for gravimetry. A Frozen in Time Lablyo −85 freeze drier was used to freeze-dry latex samples which were then compression molded using a PixMax IT91000 heat press.

### Materials

*n*-Butyl acrylate, ≥ 99%, contains 10–60 *ppm* monomethyl ether hydroquinone (MEHQ) as inhibitor, methyl methacrylate, 99%, contains ≥30 *ppm* MEHQ as inhibitor, methacrylic acid (contains 250 ppm MEHQ as inhibitor, 99%), Brij L23 solution (30% (w/v) in H_2_O), ammonium persulfate (reagent grade 98%), 4-styrenesulfonic acid sodium salt, aluminum oxide activated (neutral, Brockmann I), 2-ethylhexyl thioglycolate (>95.0%) and sodium chloride, ≥99%, were purchased from Sigma-Aldrich. Acrylic acid, 98%, extra pure, stabilized was purchased from Acros Organics. Basic activated aluminum oxide was purchased from Fluka. Dowfax 2A1, 46% active ingredient in water, was donated by the Dow Chemical Company. 2,2′-azobis[2-methyl-*N*-(2-hydroxyethyl)propionamide] (VA-086) was supplied by Wako Chemicals GmbH and was recrystallized from methanol prior to use. Styrene (contains 10–15 ppm 4 *tert*-butyl-catechol, 99%) and sodium hydrogen carbonate (99%) were purchased from Alfa Aesar Chemicals. Lakeland PAE 136 was a gift from Lakeland Laboratories. *n*-Butyl acrylate, methyl methacrylate and styrene were filtered through a short column of basic activated aluminum oxide to remove inhibitors prior to use. Methacrylic acid and acrylic acid were filtered through a short column of neutral aluminum oxide to remove inhibitors prior to use. All other chemicals were used as received. Deionized water was used in all reactions, latex analysis, and THz-TDS.

### Methods

#### Latex Synthesis

All reactions were carried out in a 250 ml double walled glass reactor, equipped with an external circulating heating bath, a Teflon anchor type stirrer fitted around 2 cm from the bottom of the reactor vessel, a condenser, and a temperature probe. Samples (1 ml) were taken throughout each reaction via a degassed syringe to analyze monomer conversion and particle size. Latex 1 was synthesized using batch emulsion polymerization and Latexes 2 and 3 were synthesized using semibatch emulsion polymerizations.

**Latex 1:** The low solids content, *SC*, “medium *T*_*g*_” latex is comprised of poly(butyl acrylate-*co*-methyl methacrylate-*co*-acrylic acid) stabilized by the ionic surfact Dowfax 2A1, which was synthesized as follows. Dowfax 2A1 surfactant solution (0.7500 g) was diluted with deionized water (177.34 g). An aqueous solution of VA-086 (0.2249 g in 14.7301 g H_2_O) was prepared. A mixture of BA (12.85 g), MMA (18.18 g), and AA (0.31 g) was prepared. The entire surfactant solution along with monomer solution (34 ml) was added to the reactor. The reactor and initiator solution were degassed for 60 min while stirring at 275 rpm. The reactor was then heated to 85^◦^ C and a shot of initiator solution (15 ml) was added to begin the polymerization. The total reaction time was 180 min. The latex was collected by hot filtering through 200 μm mesh as the reactor was drained. The latex had a final monomer conversion of > 98% calculated by gravimetry, an average hydrodynamic diameter, *d*_*z*_, of 164 *nm* with a polydispersity index, *PDI*, of 18.0%. The final *SC* was 17.1 wt % and the glass transition temperature, *T*_*g*_, measured by dynamic scanning calorimetry, DSC, was 18^◦^*C* using the half-height analysis method.

**Latex 2:** The high *SC*, “high *T*_*g*_” latex was comprised of poly(styrene-*co*-methacrylic acid) stabilized by a mixture of anionic and nonionic surfactants, which was synthesized as follows. Lakeland PAE 136, 0.32 g, in water, 105 g, was added to the reactor with 4-styrenesulfonic acid sodium salt, 0.08 g. This was degassed, via nitrogen bubbling, for 30 min, together with each of the following in separate round-bottom flasks: monomer mixture (styrene:methacrylic acid 97:3 weight ratio, 95 g), surfactant mixture (brij L23 30% w/v in H_2_O, 9.2 g and water, 5.6 g), and initiator solution (ammonium persulfate, 0.2 g, and water, 8 g). Once degassed, the monomer charge (8 g) was added to the reaction vessel and stirred, 220 rpm, for 10 min. The vessel was heated to 70 °C before the initiator charge (8 ml) was added at *t* = 0. Feed 1 (monomer, 25.962 ml hr^–1^) and feed 2 (surfactant mixture, 4.10 ml hr^–1^) began at *t* = 20 min and fed for 3 hr. The reaction mixture was stirred throughout at 220 rpm and heated via an inbuilt water jacket at 70 °C. Samples were taken throughout the reaction to monitor the conversion and particle size. The total reaction time was 4 hr and 20 min. The latex was collected by hot filtering through 200 μm mesh as the reactor was drained. The latex had a final monomer conversion of 95% calculated by gravimetry, an average *d*_*z*_ of 204 nm with a *PDI* of 4.3%. The final *SC* was 39.9 wt % and the *T*_*g*_ was 103 °C using the half-height analysis method.

**Latex 3:** The high *SC*, “low *T*_*g*_” latex was comprised of poly(butyl acrylate-*co*-methyl methacrylate-*co*-acrylic acid) stabilized by a mixture of anionic and nonionic surfactants, which was synthesized as follows. Lakeland PAE 136, 0.32 g, in water, 111 g, was added to the reactor. This was degassed, via nitrogen bubbling, for 30 min, together with each of the following in separate round-bottom flasks: monomer mixture (butyl acrylate: methyl methacrylate: acrylic acid: 2-ethylhexyl thioglycolate 92.70:4.92:2.33:0.05 weight ratio, 96 g), surfactant mixture (brij L23 30% w/v in H_2_O, 9.2 g, and water, 6.7 g), and initiator solution (ammonium persulfate, 0.24 g, and water, 11.5 g). The reaction followed the same procedure as the synthesis of Latex 2 except the feeds began at 30 min, fed for 3 h, and the total reaction time was 5 h and 30 min. Again, samples were taken throughout the reaction to monitor the conversion and particle size. The latex had a final monomer conversion of > 99% calculated by gravimetry, an average *d*_*z*_ of 211 nm with a *PDI* of 7.2%. The final *SC* was 40.4 wt % and the *T*_*g*_ was −50 °C using the half-height analysis method.

#### Latex Characterization

*SC*: Latex, either the original *SC* or diluted with distilled water, was weighed into an aluminum pan, *P*_*w*_, of known mass, *P*, and left to dry for 12 *h* at room temperature, followed by drying at 105 °C in a vacuum oven for 12 *h*. The dry mass of the pan and the remaining sample were recorded, *P*_*d*_. The *SC* was calculated using eq 1.
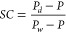
1

*d*_*z*_ and *PDI*: A sample of latex (approximately 5 mg of polymer) was diluted in distilled water (approximately 7 ml). A disposable cuvette was rinsed with distilled water filtered through a 0.22 μm hydrophilic PTFE syringe filter before filling. The measurements (settings: backscatter 175 °C, 4 min equilibration time at 25 °C, constant refractive index of water, 1.3303) were recorded in triplicate and the mean was reported.

*T*_*g*_: Approximately 10 mg of latex was dried in a Tzero Hermetic Aluminum pan (temperature range −180 °C to 600 °C, 40 μL capacity) and sealed with a Tzero Hermetic lid when using the TA Instruments DSC2500. A 40 μL aluminum pan and lid sealed the sample inside when using the Mettler-Toledo DSC. Three heating and cooling cycles were carried out at a rate of 10 K min^–1^ under air between −50 and 90 °C (Latex 1), 60 and 140 °C (Latex 2) and −150 and 50 °C (Latex 3). The *T*_*g*_ was determined using the midpoint at half height from the final heating ramp.

#### Single Point THz-TDS

Latex (1 ml) or a solid sample (100% *SC*, obtained by freezing-drying the original latex and subsequent compression molding at 150 °C for 60 s) was deposited atop the quartz imaging window and restrained within a constant area by a circular hollow structure to obtain a homogeneously layered sample, greater than 1500 μm in height, Figure S3. Due to the poor contact with the quartz window of the 100% *SC* samples, particularly of Latex 2, the high *T*_*g*_ rigid sample, the THz-TDS of these samples was performed in a transmission setup, all other single point measurements were conducted in reflection mode. In transmission mode, the optical properties of the sample are extracted using the fast Fourier transform (FFT) of the sample and reference signal only, no baseline is required. 30 pulses were recorded immediately with the THz (∼7.5 s total) after deposition. The measurement was recorded in triplicate (reflection mode) or averaged over ten repeats (transmission mode) and the refractive index, ***n***, ± 1 standard deviation, was plotted against the SC of the respective sample to create calibration lines.

All samples were measured at ambient room temperature unless otherwise stated. Where the temperature does vary, samples were cooled in an ice bath or heated in a water bath immediately before deposition. The temperature was recorded of the latex when deposited on the imaging window using a type K thermocouple.

#### Hydration Maps

A droplet of latex (0.05 ml) was deposited, using a micropipette, onto the imaging window and dried at ambient room temperature, Figure S3. Maps of ***n*** in 2D space, were acquired by applying the same setup used for the single point THz-TDS on a motorized x-y imaging stage. The speed of the stage permitted an acquisition rate of a 15 × 15-pixel image approximately every 2 min, with a resolution of 1 mm in both directions. These images were later resized via interpolation into 30 × 30 pixel figures. A LOWESS regression method, previously developed by Chen et al.,^33^ was used to account for any THz signal fluctuations and spatial phase variation due to variations in the imaging window thickness. The method required a reference measurement for every point in the image and a baseline to be acquired at the center window position as discussed in the results and discussion.

A weighted average of the water content, *WWA*, of the latex droplets was calculated, eq 2, by applying a 2D Gaussian filter (with a SD of σ = 4 to fit the shape and width of the droplet) on the matrix centered on the pixel with the highest water content in the first map created. The *w*_*i*_ corresponds to the weight of the pixel, *i*, extracted from the Gaussian filter. Both the *WWA* and the maximum water content from each hydration map are reported.
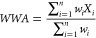
2

#### Film Formation Gravimetry

A droplet of latex (0.05 ml) was deposited onto a glass slide on an analytical balance to coincide with the time when a latex droplet was deposited onto the imaging window to measure THz-TDS. This was to account for the samples experiencing the same environmental conditions during drying. The mass of the droplet was recorded to coincide with the acquisition of each hydration map.

## Results and Discussion

The sensitivity of THz radiation to water has been utilized to analyze the drying and film formation of water-based polymer latexes. The evaporation of water from sessile droplets containing the polymer colloids was tracked. A reflection optical setup was used to obtain refractive indexes, *n*, using THz-TDS, which we at first approximation correlated linearly to a sample’s solids content, *SC*, to produce 2D_*xy*_ hydration maps.

Three latexes were used to showcase the broad range of applications of the technique. Latex 1, a random copolymer of poly[(*n*-butyl acrylate)-*co*-(methyl methacrylate)-*co*-(acrylic acid)] had a *SC* of 17.1 wt % and a *T*_*g*_ just below room temperature. Latex 2, poly(styrene-*co*-methacrylic acid) and 3, poly[(*n*-butyl acrylate)-*co*-(methyl methacrylate)-*co*-(acrylic acid)], had a higher *SC* of 40 wt % and high and low *T*_*g*_’s, respectively. The range of *T*_*g*_’s is used to demonstrate how the technique performs when drying occurs above, typically producing transparent, homogeneous films, and below, producing opaque, cracked films, the minimum film formation temperature (MFFT). The average hydrodynamic diameter, *d*_*z*_, was relatively similar for all three latexes. These properties are detailed in Table 1.

**Table 1:** Solids Content, *SC*, Average Hydrodynamic Diameter, *d*_*z*_, Particle Size Dispersity, *PDI*, and Glass Transition Temperature, *T*_*g*_, for the Latexes Used in This Work

Latex	*SC*/wt %	*d*_*z*_/nm	*PDI*/%	*T*_*g*_/°C
Latex 1	17.1	164	18.0	18
Latex 2	41.7	204	4.3	103
Latex 3	39.5	211	7.2	–50

### Single Point THz-TDS

**Figure 2:**
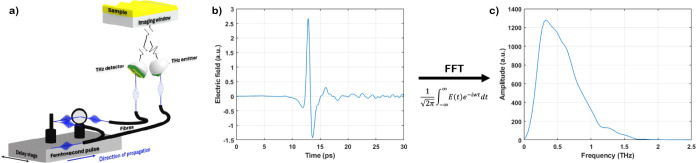
(a) Schematic representation of a THz reflection setup. (b) Measured THz time-domain signal. (c) Fast Fourier transform (FFT) of the measured THz signal in the frequency domain.

The reflection setup used for single point THz measurements is depicted in Figure 2a and a typical THz pulse recorded using this setup is shown in Figure 2b. This signal is transformed from the time-domain to the frequency-domain, Figure 2c, using a fast Fourier transform (FFT). This provides amplitude and phase information which allows the direct calculation of the complex refractive index, ***n***, of the sample as long as a known reference, in this case air, and baseline are also measured. The mathematical process to obtain the sample ***n*** and therefore the real refractive index, *n*, is explained in detail in the Supporting Information.

**Figure 3:**
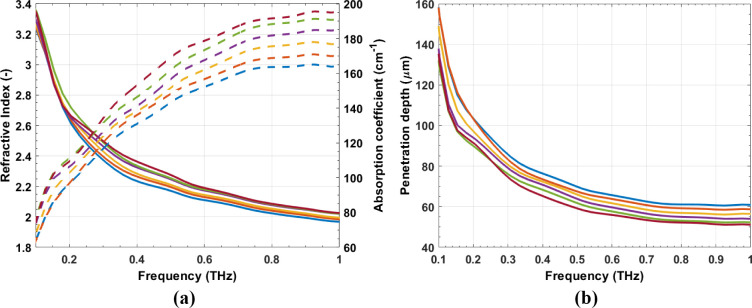
Latex 1, at *SC* of 0 (red), 3.42 (green), 6.86 (purple), 10.28 (yellow), 13.70 (orange) and 17.13 (blue) wt %, was analyzed using THz-TDS. (a) *n* (—) and absorption coefficient (- - -) over a range of THz frequencies. (b) Penetration depth of the pulse as a function of THz frequency. The data obtained for the calibration line was extracted at a frequency of 0.62 THz for optimal SNR.

Despite obtaining the *n* for a range of frequencies, 0.2–1 THz, only one *n* is reported for each sample. The frequency chosen, 0.62 THz, gave the best signal-to-noise ratio (SNR) when comparing samples of different *SC* of Latex 1, obtained by dilution with distilled water, Figure 3a. This frequency was used for all other samples to allow comparisons between hydration maps of different latexes. The frequency chosen for the analysis can be tailored to the sample. For example if a sample absorbs too much at 0.62 THz, it might be better to choose a different frequency depending on the sample properties. Figure 3b shows the penetration depth of the THz as a function of frequency. In general the lower solids contents have a higher absorption coefficient and therefore lower penetration depth (the reciprocal of the absorption coefficient). This is expected as water has a large absorption coefficient in the range of scanned THz frequencies.^34^ As can be seen from Figure 3b the penetration depth is around 55–65 μm at 0.62 THz. This means the *n* calculated is an average of the bottom 60 μm of the sample. If the drying process of the sample has a Peclet number, *Pe*, considerably less than unity, meaning the diffusional motion of the latex particles is fast relatively to the radial velocity of the receding droplet surface, then we can assume the measured data averages across the entire height of the droplet.^14,35^ If, however, *Pe* > 1 our average becomes inaccurate as there will be particle accumulation at the drying front forming a skin at the top of the drying droplet which results in a particle concentration gradient and subsequently a water concentration gradient.

**Figure 4:**
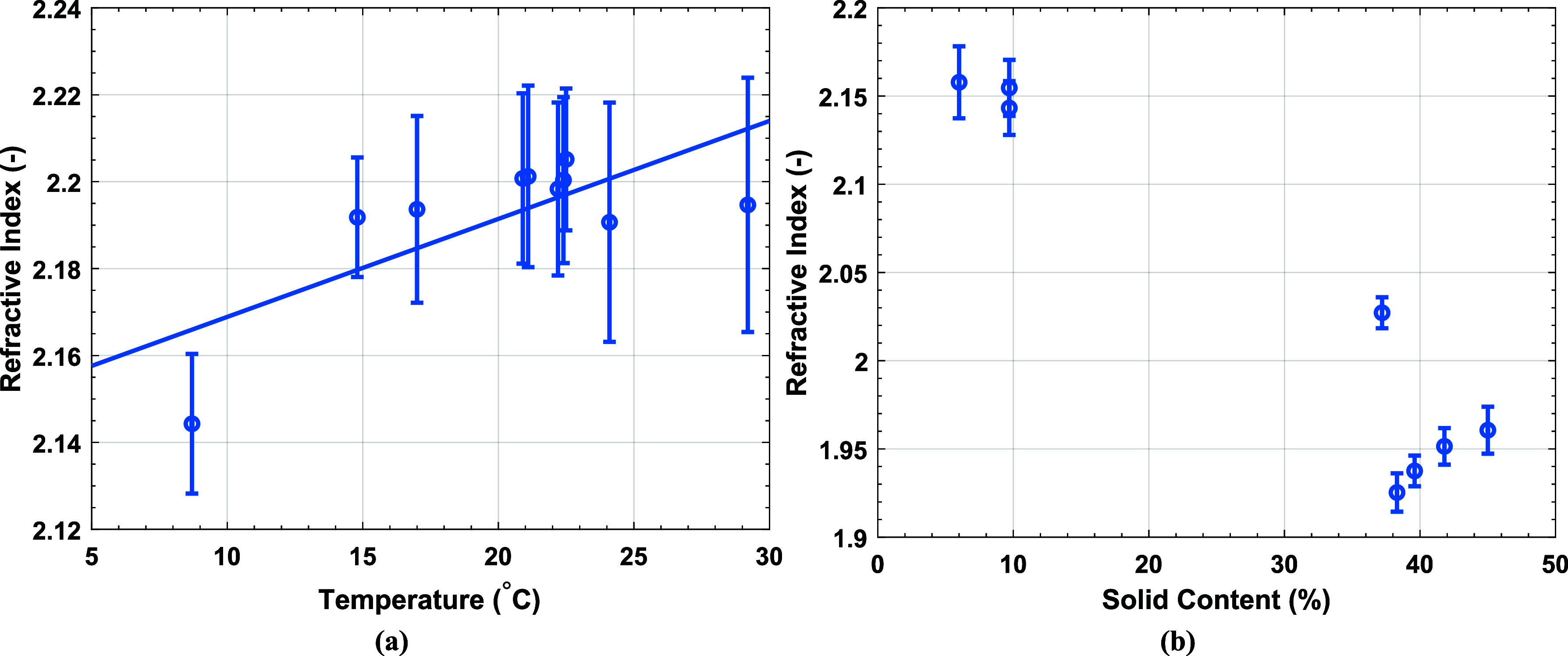
(a) *n* of distilled water at a frequency of 0.62 THz against temperature with a linear fit, *r*^2^ = 0.53. (b) *n* of latexes of various polymer chemical compositions with different *SC* show no correlation.

The setup used for these experiments did not have a temperature-controlled platform so the effect of small temperature fluctuations on the extracted *n* was investigated. Figure 4a demonstrates a positive correlation between temperature and the *n* of water, also confirmed by Zhou and coworkers.^34^ This effect was negated in our experiments in two ways. First, the measurements were always taken in a short time period where the temperature fluctuations of the room the measurements were conducted in were low (±0.5^◦^ C). Additionally, the *n* measured was always normalized with respect to the *n* of water, measured in the same session, making it possible to compare measurements made on different days under slightly different environmental conditions.

A variety of latexes were used with varied *SC*, polymer composition, *d*_*z*_ and *T*_*g*_, detailed in the Supporting Information, to determine if there was a general trend between *SC* and extracted *n* from THz-TDS. The results of this can be seen in Figure 4b and clearly show there is no clear trend. It is not obvious if this is due to the difference in polymer composition or *d*_*z*_ and *PDI* of the individual samples. Therefore, separate calibration lines of *n* against *SC* were created for Latexes 1–3, which were then used to produce the hydration maps shown later in this work.

### Refractive Index Calibration

A calibration of *n*, extracted from THz-TDS, against *SC* was created for Latexes 1–3. The varied *SC* were obtained by diluting the original latexes with distilled water. *SC* above the original synthesis levels were difficult to obtain without causing particle flocculation or coagulation. A 100% *SC* sample was obtained by freezing-drying the original latex and subsequently compression molding it at a high temperature.

**Figure 5:**
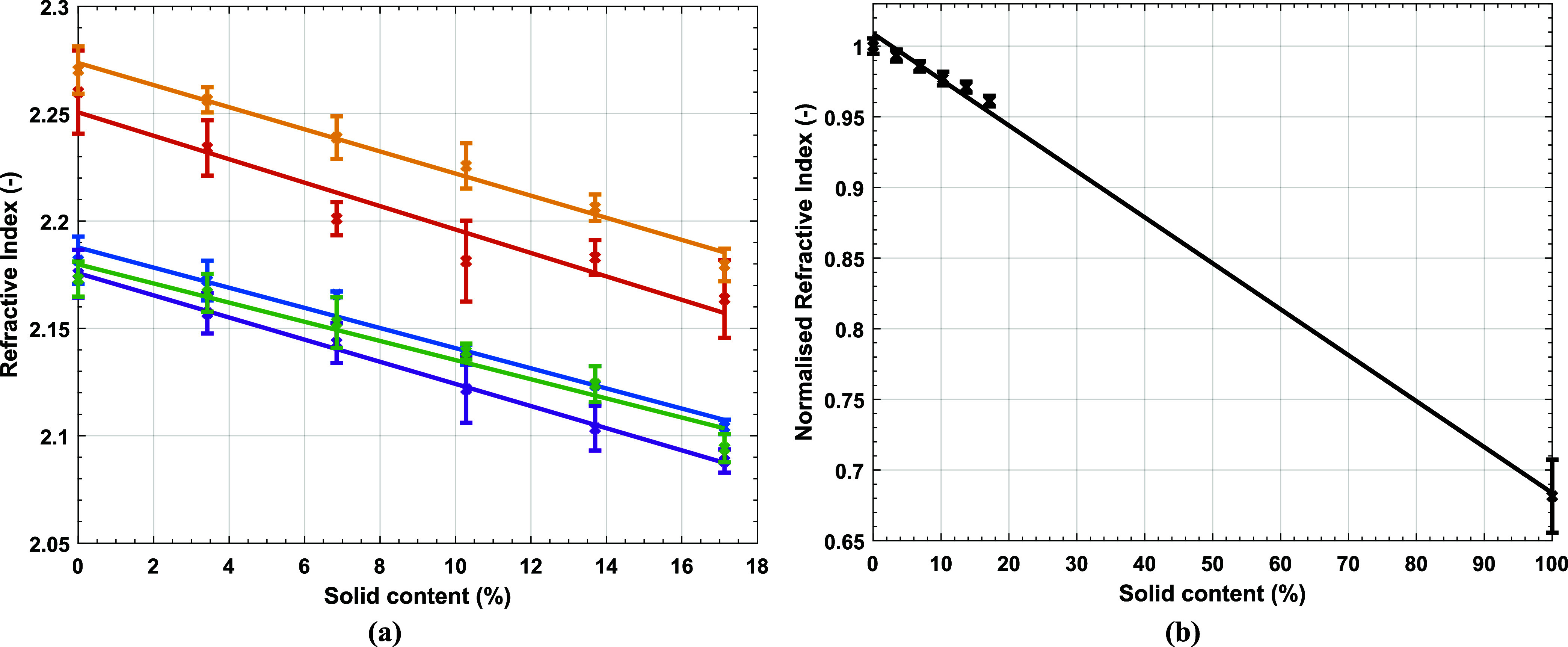
(a) Refractive index, *n*, extracted by THz-TDS at 0.62 Hz, against *SC* of Latex 1 with a linear fit where the coefficient of determination, *r*^2^, is above 0.92 in all repeated measurements. Each color represents a different measurement session under slightly different environmental conditions. The measurements of only the latex are shown here. (b) The average of the *n*, normalized against the *n* of distilled water under the same environmental conditions, against *SC* of Latex 1. The linear fit has an *r*^2^ of 0.9973. The measurements of the latex and dry polymer are shown here.

**Figure 6:**
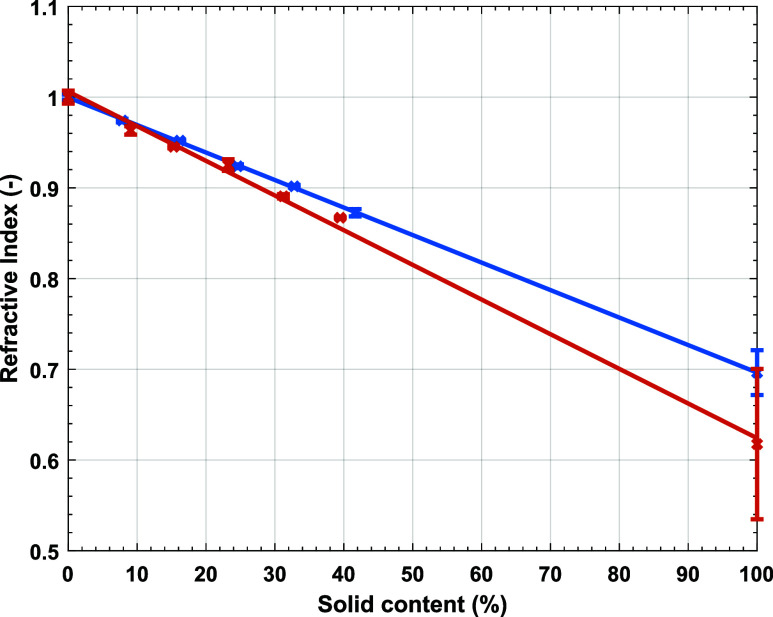
Normalized *n* against *SC* for high *T*_*g*_, Latex 2 (orange circle), and low *T*_*g*_, Latex 3 (blue circle). Each measurement was repeated seven times and an average of the normalized *n*, ± 1 SD, is shown.

The calibration for Latex 1 is shown in Figure 5. The calibration was repeated on five separate days where the environmental conditions varied slightly, 20 < *T* < 25 °C and approximately 60% relative humidity. Figure 5a, shows how linear calibration lines, obtained via a linear least-squares fitting method, were a reasonable fit to the data but were affected by the fluctuations and makes accurate comparison between repeats difficult. There is a marked vertical shift between data sets attributed to the fluctuation in environmental conditions such as humidity,^36^ temperature,^34^ laser output power and baseline measurements. The gradients of the linear fits, however, show good agreement, with a coefficient of variation of 8.3%. We therefore normalize *n* to the *n* of distilled water under the same measurement conditions (See Figure 5b). However, due to the low *SC* of the synthesized latex, there are many *SC* unable to be measured. The use of a linear correlation was further investigated using the higher *SC* latexes, Latex 2 and 3, which continued to show a linear relationship (see Figure 6). The intercept on the vertical axis of the linear fit always resulted in a value close to 1, since the *n* was normalized relative to the *n* of water, which is at 0% *SC*. The gradient showed slight variations of −0.0033, −0.0038, and −0.0030 for Latexes 1, 2, and 3 respectively. This is likely due the variation in *d*_*z*_, *PDI* and polymer composition resulting in differences in the measured THz pulses due to scattering. The linear fits presented for the three latexes in this work are relatively similar suggesting there may be the possibility of a universal calibration which accounts for the polymer *n* and the latex particles *d*_*Z*_ and *PDI*.

### 2D_*xy*_ Hydration Maps

The experimental setup used for single-point measurements was used on a motorized x-y stage to produce 2D_*xy*_ maps of *n*, which were then converted to hydration maps using the calibration lines produced in Figures 5 and 6. Since there is only one THz detector in this setup there is an acquisition period of 2 *min* for each 15 × 15 data array. This could result in changes in water evaporation between the start and end of the acquisition. In the case of the latex droplets dried in this work, the drying time was at least 30 *min*, Latex 2, or greater (60 *min* for Latex 1 and 3), so the effect was minimal but should be considered for faster drying samples. It should be pointed out that this is not an inherent limitation of the technique but only of current THz technology. The current state-of-the-art THz imaging systems have been able to obtain similar information in a few seconds^37,38^ and show potential for even further speed improvements.

In the following analysis, we assume that the THz response from water remains the same during all stages of film formation. It is unclear if this stops being the case when the interstitial spaces between particles become so small that we get overlapping electrostatic double layers and surfactant/steric layer interpenetration, hereby potentially altering the spectroscopic response of the water molecules (state 1 to state 2, Figure 1). Once a droplet was dry, the signal-to-noise ratio decreased significantly due to the lack of absorption of the THz pulse and it was difficult to determine the difference between areas where there was dried latex and areas where there was just the quartz window. To ensure only the latex droplet is shown in the *n* and hydration maps, the first maps recorded when the droplet was deposited, where the *SC* was lowest and water content highest, were used to determine the pixels shown in the subsequent images. For the latexes used in this work this method worked well as they did not dewet and the droplets pinned on the quartz substrate after initial deposition. For latexes with less favorable interfacial tensions with quartz this data processing method may need adaptation to also track the pinning lines.

**Figure 7:**
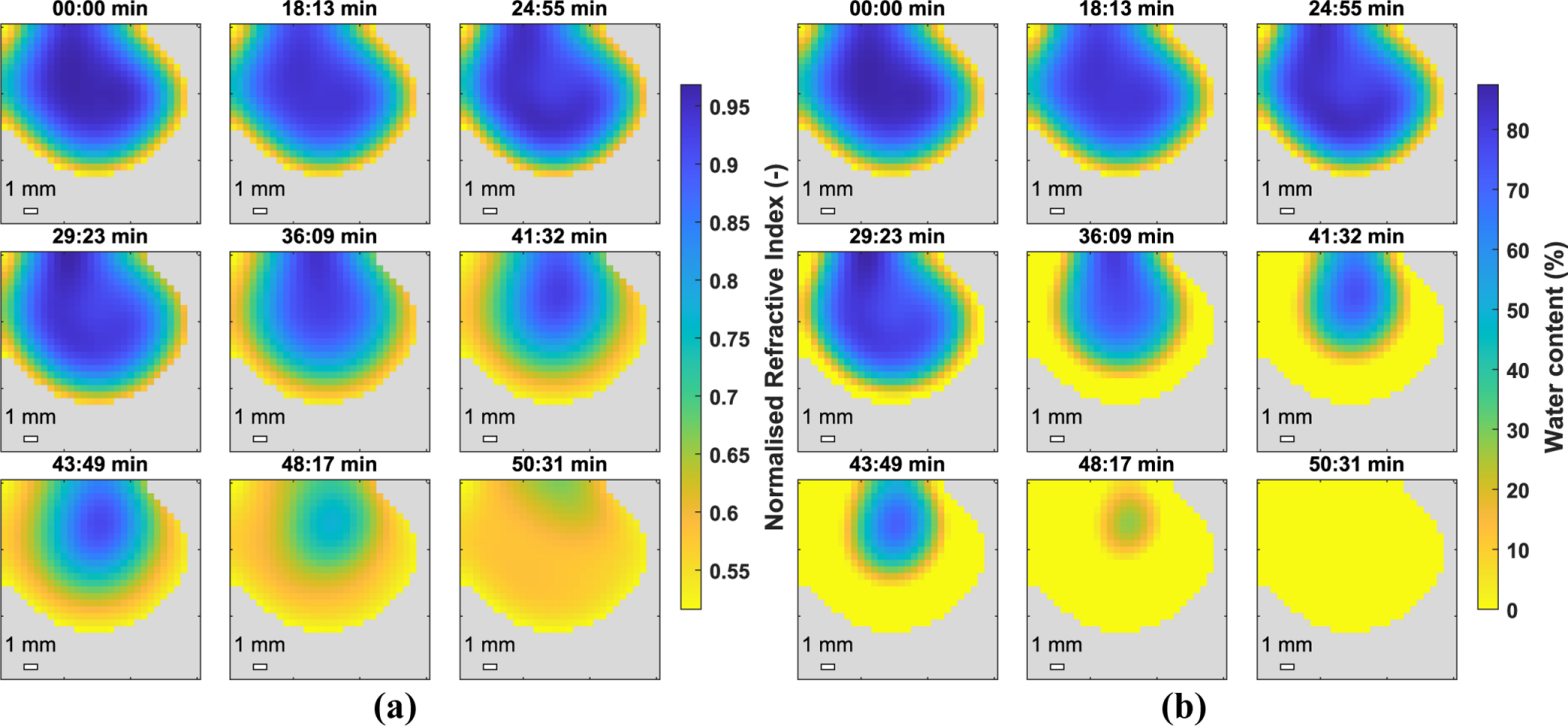
2D_*xy*_ normalized *n* (a) and water content (b) of a droplet of Latex 1. Film formation was very slow for the first 30 min so some of the early maps are omitted (21 data arrays were recorded in total) to demonstrate the faster drying rate at the end of the process.

**Figure 8:**
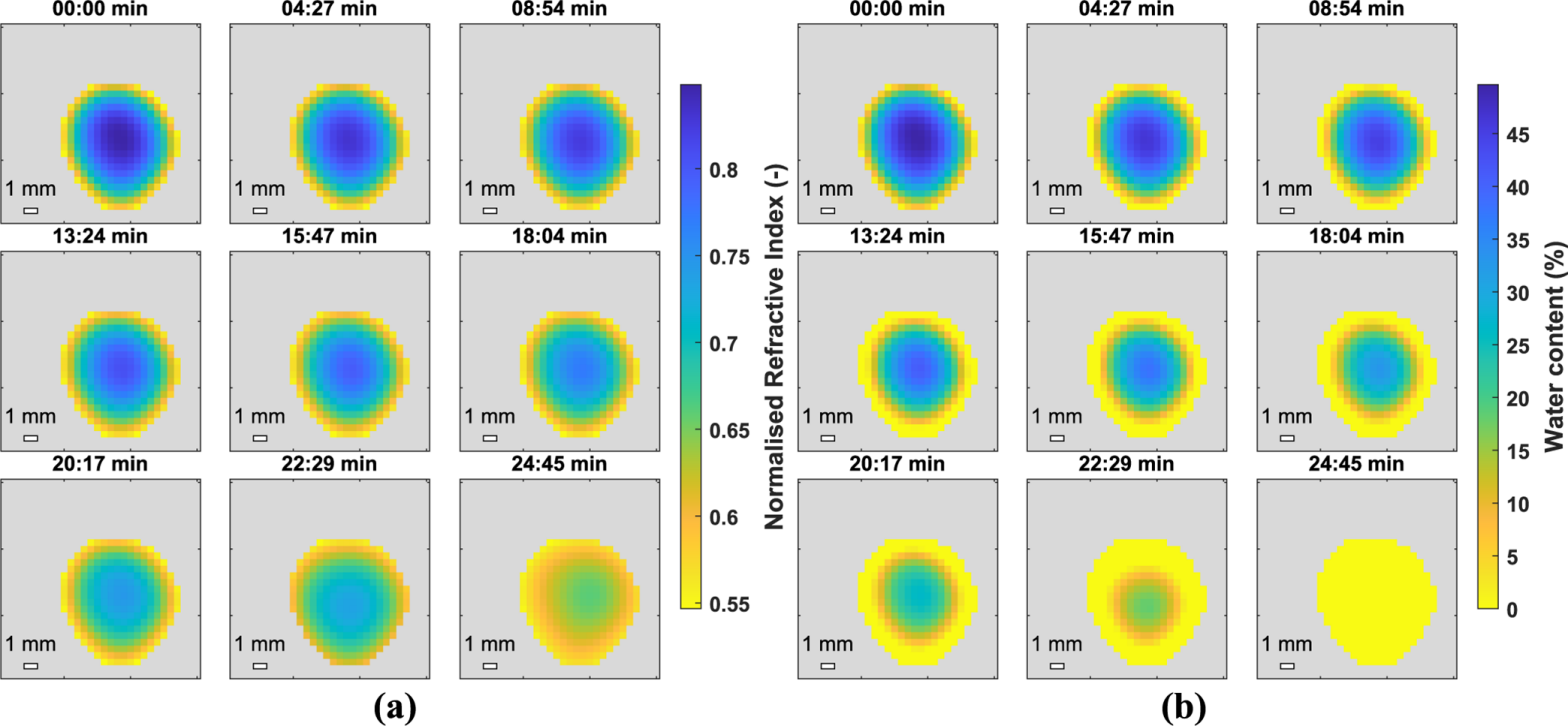
2D_*xy*_ normalized *n* (a) and water content (b) of a droplet of Latex 2.

**Figure 9:**
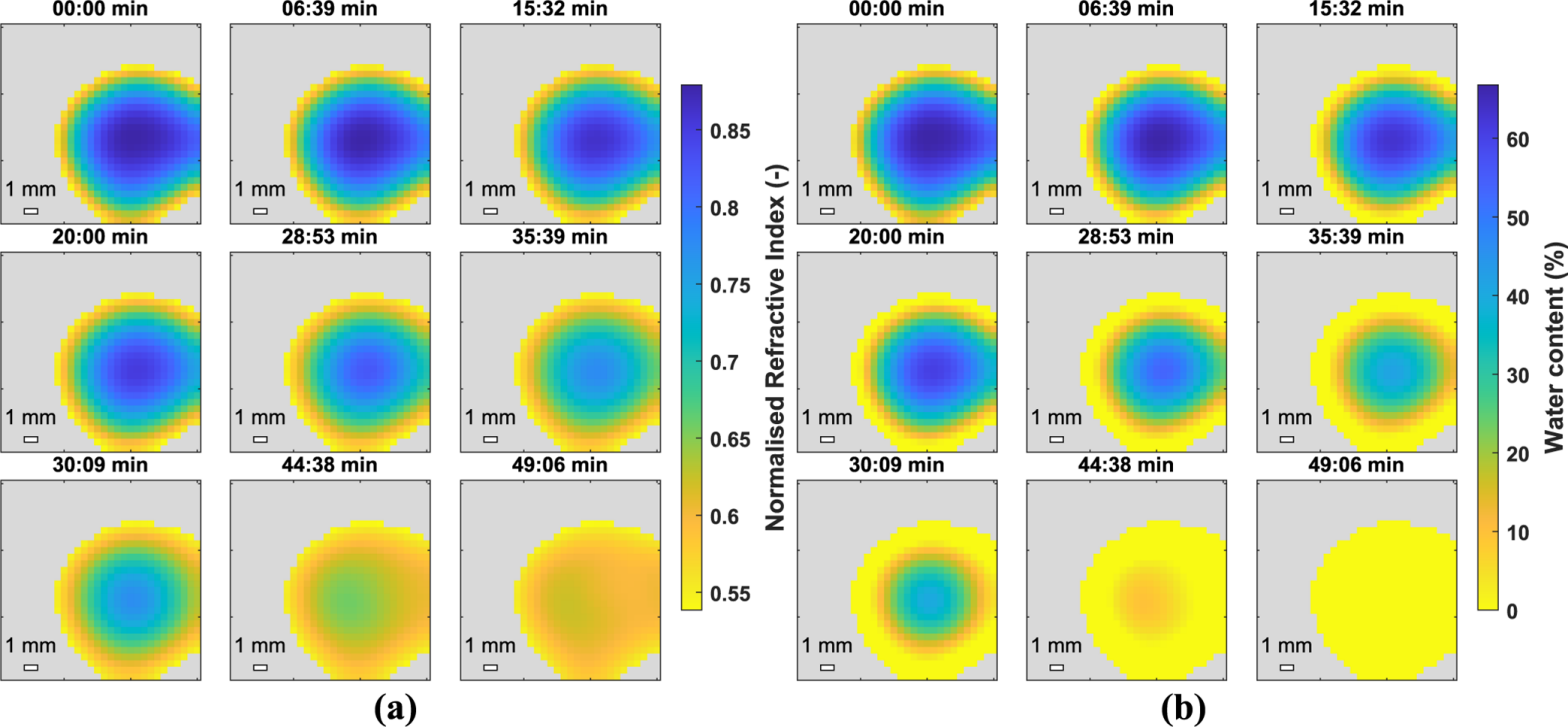
2D_*xy*_ normalized *n* (a) and water content (b) of a droplet of Latex 3.

Figure 7 shows the *n* and hydration map for a droplet of Latex 1 during film formation. It can be seen that the droplet dries at the edges first and the drying front moves inward toward the center of the droplet, as described by Routh and Russel.^7,8,12^ The droplet was not perfectly spherical, so the drying front did not move toward the center perfectly radially. Latexes 2 and 3 were analyzed using the same method and *n* and hydration maps are shown in Figures 8 and 9 respectively. Again the hydration map demonstrates how the droplet dries from the edges first with a drying front moving inward radially. The most obvious difference is the time taken to completely dry, with Latex 2 taking less than 30 *min* and Latex 3 approximately 50 *min*. This can be attributed to the difference in *T*_*g*_’s and agrees well with work reported by Carter and coworkers using GARField-NMR who attribute the slower drying of softer, lower *T*_*g*_ films, to lower polymer viscosities allowing the growth of coalesced skin layers impeding water evaporation.^9^ The broad range of *T*_*g*_’s used shows the applicability of this technique to many industries such as adhesives and coatings.

### Method Verification Using Gravimetry: A Challenge

To verify THz-TDS as an accurate and precise method to monitor drying and film formation, gravimetry was performed at the same time on two separate droplets of Latex 2 and Latex 3, such that they experienced the same environmental conditions as their THz-TDS counterparts. Since the THz-TDS had an acquisition time longer than that required for gravimetry, the mass of the sample was always taken at the start of the THz-TDS measurement. It is difficult to compare the methods directly as gravimetry gives you the overall amount of water in the total sample, that is one value over time whereas THz-TDS gives you an array of data, 2D, over time, but only of the ca. 60 μm layer of the droplet in contact with the quartz imaging window. Therefore, the 2D array has to be converted to a single average value for comparison. This can be approached in a variety of ways. In this work we have used two different methods for comparison; first a weighted average and then a single column of pixels comparison to explore the THz-TDS method validity.

#### Weighted Average

The latex droplet shape complicates simply taking an average of all the pixels in the 2D array from THz-TDS. In hindsight, looking at a infinite flat film of drying latex would have been less problematic, because any boundary effects would have been avoided. In addition, the analyzed droplets of latex are not idea spherical caps as can clearly be seen from Figures 7–9 hereby complicating radial symmetry and thus data analysis. To account for this a weighted average, WA, was calculated by superimposing a Gaussian matrix on the hydration maps and extracting their Frobenius inner product. The values of this matrix are given by the 2D Gaussian function presented in eq 3, where σ = 4 was used. The Gaussian function was centered around the highest water content value from the first hydration map imaged for each latex and the same pixel was used as the center for all subsequent maps.
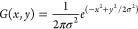
3

**Figure 10:**
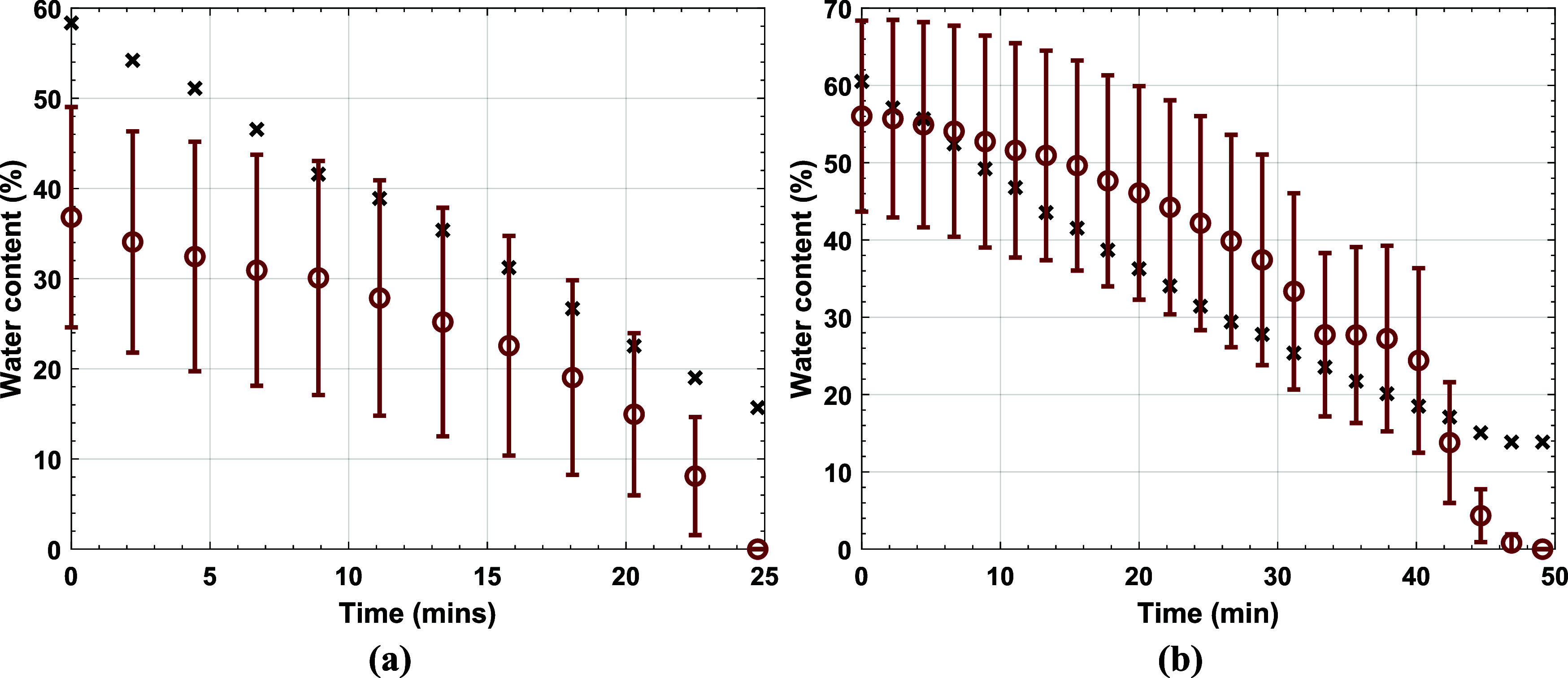
Comparing the gravimetric results (×) with the weighted average of the hydration content (red open circle) using a Gaussian filter for Latex 2 (a) and Latex 3 (b).

**Figure 11:**
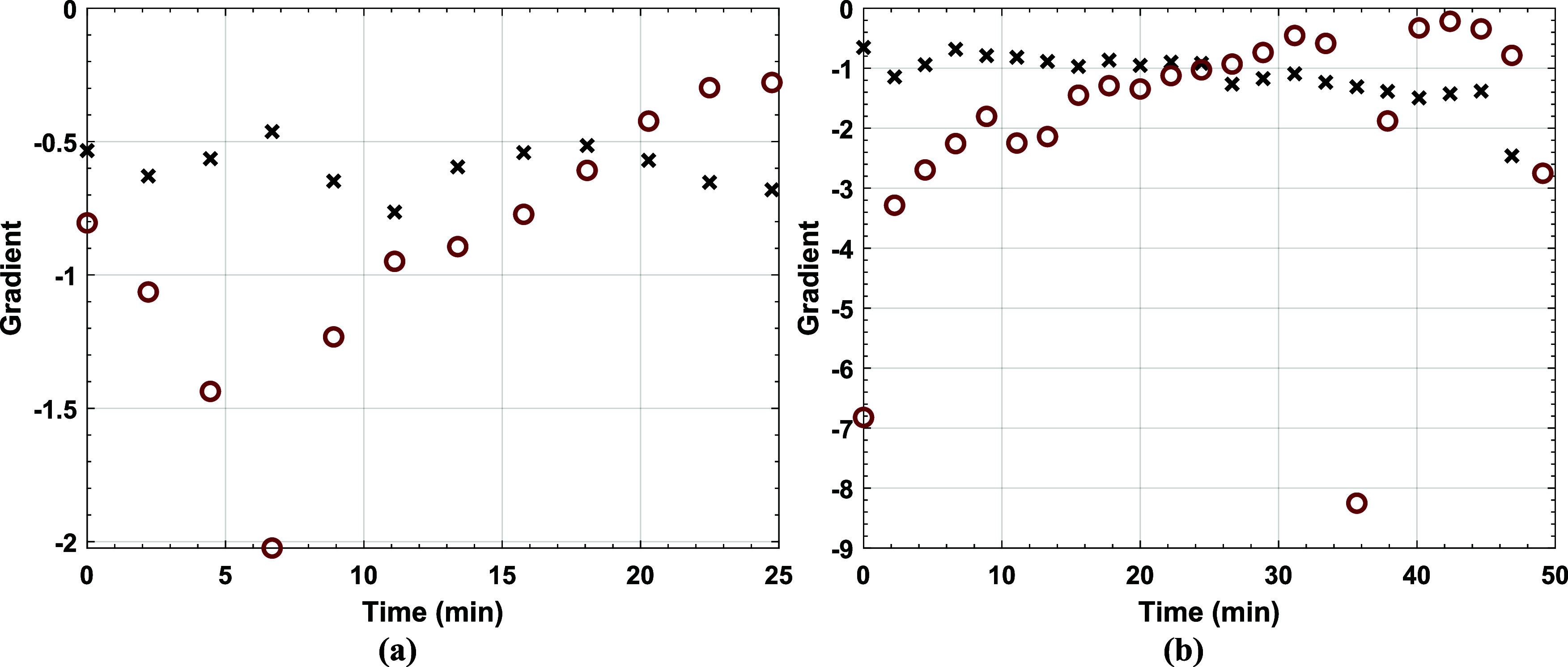
Comparing the gradient (rate of change) of the gravimetric results (×) with that of the weighted average of the hydration content (red open circle) for Latex 2 (a) and Latex (3).

Figure 10 shows the water content calculated from gravimetry and the WA from THz-TDS for Latexes 2 and 3. In general the results agree more for Latex 3, Figure 10b, than for Latex 2, 10a. There are two main reasons for this. First, the Gaussian function used to calculate WA describes the entire droplet shape and as previously discussed, the THz pulse does not explore the entire droplet so this mismatch increases the error. Second, Latex 2 has a high *T*_*g*_ and is likely to crack due to the particles resistance to deformation when dried at room temperature. This creates cracks and microcracks in the dried polymer material which increased the error in the *n* measured. The rate of drying reported by gravimetry and the WA from THz-TDS was compared, Figure 11, by linearly interpolating the data reported in Figure 10 and calculating the gradient at the times corresponding to the measurement times. Again, Latex 3, Figure 11b, agrees more than Latex 2, Figure 11a. This can be attributed again to the Gaussian model used and the high *T*_*g*_ of Latex 2. Overall we can agree that the differences between gravimetry and THz data interpretation are substantial, and needs improvement.

#### Single-Column Pixel Comparison

**Figure 12:**
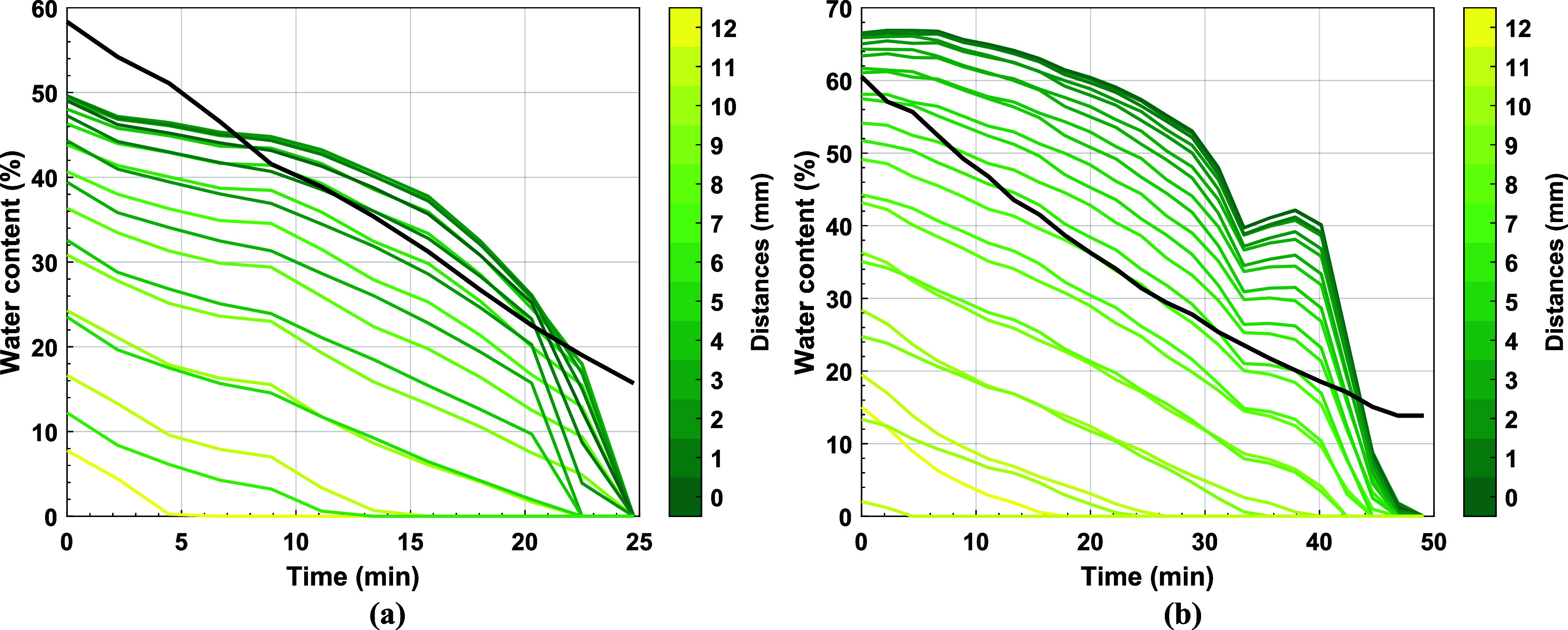
Comparing the water content extracted using gravimetry with the center column of pixels from their respective hydration map over time for Latex 2 (a) and Latex 3 (b). The distance between the pixel whose hydration content is measured from the central pixel is given in the color-bar.

To avoid the error produced when calculating the WA, the water content of each pixel was compared overtime. A line of pixels through the center of the droplet, determined from the highest water content pixel in the first hydration map measured, was chosen as a representation of the entire hydration map. Figure 12, shows the comparison of the water content over time as a function of pixel distance from the center of the droplet for Latex 2, Figure 12a and Latex 3, Figure 12b. Similar drying patterns are observed for both latexes in the THz-TDS. We can see from both THz data sets that the loss of water initially seems to occur at a lower rate for the central parts of the droplet in comparison to the gravimetric water loss. If correct this could indicate a concentration gradient of polymer latex particles, radially, promoting a so-called coffee ring effect possibly challenging the validity of low local Peclet numbers.

### Conclusion

This paper presents the use of THz-TDS to analyze the drying process and film formation of water-based polymer colloids and produces hydration maps to monitor water content as the latex dries. Currently, a calibration of *SC* against *n*, extracted from THz-TDS, is required to produce the hydration maps. We approached this crudely by assuming linearity and by normalizing the data against *n* of water. The accuracy of this approach should be tested with a wider range of *SC*s of the polymer dispersions. Also the influence of variation in the chemical characteristics of the polymer dispersion needs to be explored in more detail. Future work should also focus on altering the *d*_*z*_ and *PDI* of the latex particles to determine if there is the possibility of a universal calibration. We selected a single frequency to analyze our data, but obviously one could argue to use all data of a wider frequency window. The frequencies used in the analysis can be selected to highlight the properties of interest for a particular sample and depend on sample parameters such as film thickness or droplet height and difference in *n* between the water and polymer particles. In this work, the technique has shown promise for polymer latexes with varying *T*_*g*_’s, suitable for a wide range of applications. In general, the ability to produce hydration maps is valuable. More work needs to be done to correlate the measured water content with gravimetric data. Simplifying the experimental setup to initially look at flat drying films, rather than droplets, may bring outcome.

This technique is not, in theory, limited to the study of latex film formation and could be adapted to other water-based systems where the properties of the resulting film or coating rely heavily on the drying process. We hope to have shown that this technique is promising and can fill the dimensional gap and be used in conjunction with other techniques, such as optical coherence tomography and GARField NMR, to continue the in-depth study of film formation processes. We acknowledge that more work is needed to validate its accuracy.

## Supporting Information Available

Information on the polymer latexes, including monomer conversion and particle size data against time of the emulsion polymerization reactions, and polymer latex DSC data; details of the THz-TDS setup together with the approach on how to calculate the refractive index from the THz-TDS data (PDF)
