# Supplementary material for: Developing Hydration Maps of Polymer Latex Film Formation Using Terahertz Time-Domain Spectroscopy
Source: Langmuir. 2024 Nov 13;40(47):25023–33. doi: 10.1021/acs.langmuir.4c03103 (PMC11603779; doi:10.1021/acs.langmuir.4c03103)
Supplement: Supplementary file 1 — la4c03103_si_001.pdf [file la4c03103_si_001.pdf]

Electronic Supplementary Information Developing Hydration Maps of Polymer Latex Film Formation using Terahertz Time-Domain Spectroscopy

Gonçalo Costa,[†,‡] Emily M. Brogden,[¶,‡] Jacob J. Young,[†] Huiliang Ou,[†] Arturo I. Hernandez-Serrano,[†] Rayko I. Stantchev,[§,†] Stefan A. F. Bon,[*,¶] and Emma Pickwell-MacPherson[*,†]

†*Department of Physics, University of Warwick, Gibbet Hill Road, Coventry, CV4 7AL, UK*

‡*These two authors contributed equally to this work*

¶*Department of Chemistry, University of Warwick, Gibbet Hill Road, Coventry, CV4 7AL, UK*

§*Department of Physics, National Sun Yat-sen University, Kaohsiung, Taiwan*

E-mail: S.Bon@warwick.ac.uk; E.Macpherson@warwick.ac.uk

List of Figures

S1 Latex synthesis kinetics and average particle size. (a) $\rho_{M,inst}$ and $\rho_{M,cum}$ against time, (b) d_z against time, (c) PDI against time and (d) d_z against $\sqrt[3]{\rho_{M,cum}}$. 4

S2 DSC thermograms with three heating (●, ●, ●) and cooling (●, ●, ●) cycles for the latexes synthesized in this work. 5

S3 (a) To develop a calibration line, 1 *ml* of latex was contained by a circular structure atop a quartz imaging window. (b) To produce 2D images of a drying sample, 0.05 ml of latex was placed atop the quartz window. The blue area shows the dilute regions yet to dry, and the transparent area shows the drying front, dried region. Cracks can be seen along the direction in which the sample is drying. (c) The K15 THz spectrometer in a motorized x-y stage reflection setup at an incident angle of 30°. 6

S4 Schematic representation of a THz reflection measurement. (a) Acquisition of the sample signal. (b) Acquisition of the reference signal. The baseline signal, reflected from the lower surface of the quartz window, is present in both sample and reference signals. 7

List of Tables

S1 The solids content, SC, average hydrodynamic diameter, d_z, particle size dispersity, PDI, glass transition temperature, T_g, polymer composition with monomer weight ratios and synthesis methods for the latexes refered to in Figure 4b. *Not measured. 7

Experimental

Latex synthesis

The *SC* was used to calculate the instantaneous conversion, $p_{m,inst}$, at each time point using Equation S1, where $M_{t,sol}$ is the mass of all solid components, not including polymer, $M_{t,tot}$ is the mass of all components and $M_{t,mon}$ is the cumulative mass of monomer at the time of sampling.

$$p_{M,inst} = \left(SC - \frac{M_{t,sol}}{M_{t,tot}}\right)\left(\frac{M_{t,tot}}{M_{t,mon}}\right) \tag{S1}$$

Using $p_{M,inst}$, the cumulative conversion, $p_{M,cum}$, was then calculated using Equation S2, where M_{mon} is the total mass of the monomer used for the reaction.

$$p_{M,cum} = p_{M,inst}\left(\frac{M_{t,mon}}{M_{mon}}\right) \tag{S2}$$

$p_{M,inst}$ and $p_{M,cum}$ are reported as a function of reaction time for each latex in Figure S1.

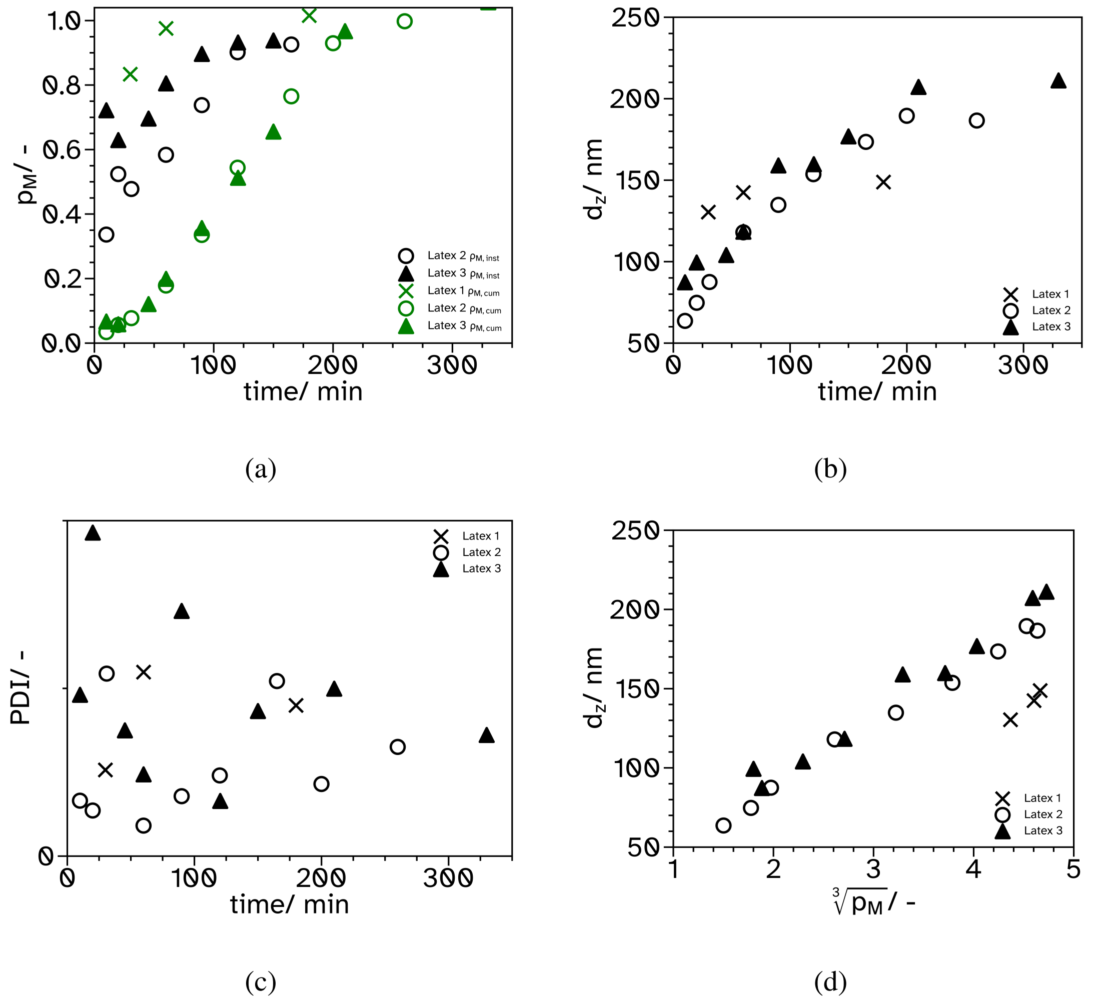

Figure S1: Latex synthesis kinetics and average particle size. (a) $\rho_{M,inst}$ and $\rho_{M,cum}$ against time, (b) d_z against time, (c) PDI against time and (d) d_z against $\sqrt[3]{\rho_{M,cum}}$.

Dynamic Scanning Calorimetry

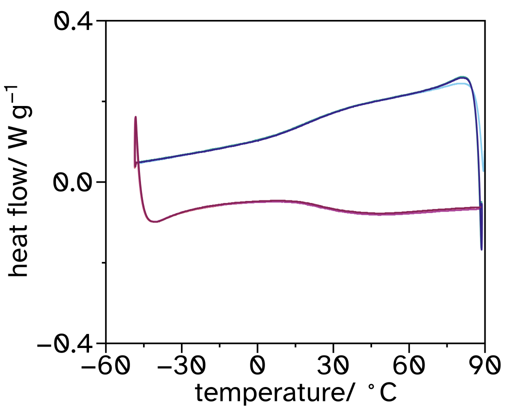

(a) Latex 1

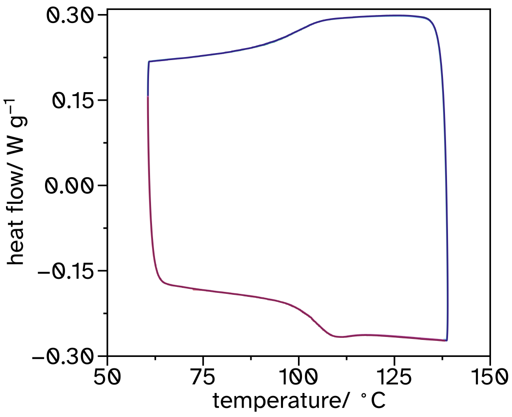

(b) Latex 2

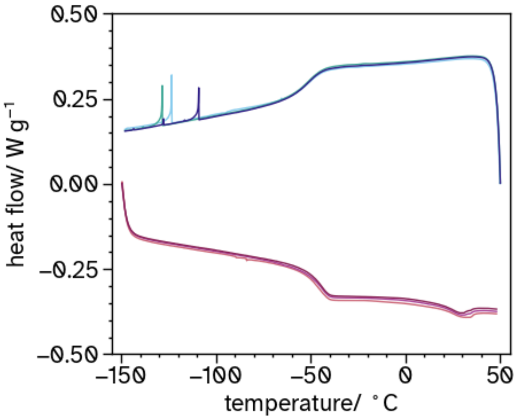

(c) Latex 3

Figure S2: DSC thermograms with three heating (●, ●, ●) and cooling (●, ●, ●) cycles for the latexes synthesized in this work.

THz-TDS Setup

h

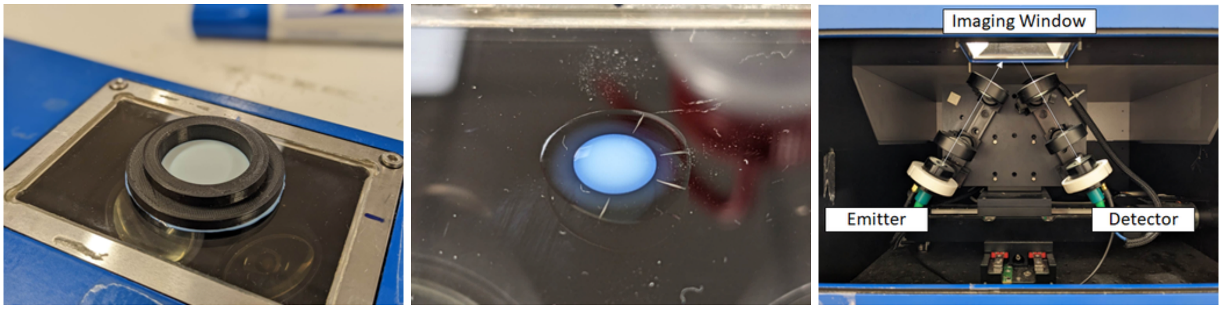

(a) (b) (c)

Figure S3: (a) To develop a calibration line, 1 *ml* of latex was contained by a circular structure atop a quartz imaging window. (b) To produce 2D images of a drying sample, 0.05 ml of latex was placed atop the quartz window. The blue area shows the dilute regions yet to dry, and the transparent area shows the drying front, dried region. Cracks can be seen along the direction in which the sample is drying. (c) The K15 THz spectrometer in a motorized x-y stage reflection setup at an incident angle of 30°.

Water content from gravimetry

The water content, WC, was calculated from gravimetric data, where m_t was the mass of the droplet at the sample time and m_0 was the original mass of the sample immediately after deposition.

$$\mathrm{WC}_t = \left(\frac{m_t}{m_0} \times 100\right) - \mathrm{SC} \tag{S3}$$

Table S1: The solids content, SC, average hydrodynamic diameter, d_z, particle size dispersity, PDI, glass transition temperature, T_g, polymer composition with monomer weight ratios and synthesis methods for the latexes refered to in Figure 4b. *Not measured.

Latex	$SC/\ wt.\%$	$d_z/\ nm$	$PDI/\ \%$	$T_g/°C$	Polymer composition	Synthesis method
Latex 4	38.4	186	4.9	106	poly(MMA-*co*-IBoMA) 9:1	semi-batch EP
Latex 5	45	174	9.3	108	poly(MMA-*co*-IBoMA) 9:1	semi-batch EP
Latex 6	6	98	8.1	-*	poly(BA-*co*-MMA) 19:1	batch EP
Latex 7	38.3	216	7.6	-49	poly(BA-*co*-MMA) 19:1	semi-batch EP
Latex 8	37.2	194	14.2	27	poly(VAc)	semi-batch EP
Latex 9	9.7	81	10.0	-*	Poly(Sty-*co*-BA) 4:6	batch pickering EP
Latex10	9.7	84	12.3	-*	Poly(Sty-*co*-BA) 4:6	batch pickering EP

Results and Discussion

Calculation of n from THz-TDS

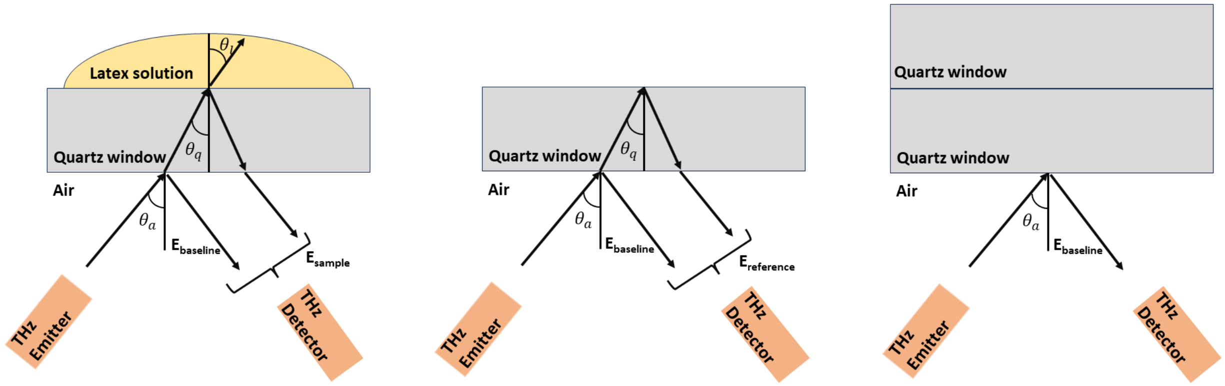

Figure S4: Schematic representation of a THz reflection measurement. (a) Acquisition of the sample signal. (b) Acquisition of the reference signal. The baseline signal, reflected from the lower surface of the quartz window, is present in both sample and reference signals.

The real refractive index of the samples was extracted by recurring to the use of the Fresnel equations for the s-polarised light,

$$M = \frac{E'_{sample}}{E'_{reference}} = \frac{\boldsymbol{n}_q \cos\theta_q - \boldsymbol{n}_l \cos\theta_l}{\boldsymbol{n}_q \cos\theta_q + \boldsymbol{n}_l \cos\theta_l} \times \frac{\boldsymbol{n}_q \cos\theta_q + \boldsymbol{n}_a \cos\theta_a}{\boldsymbol{n}_q \cos\theta_q - \boldsymbol{n}_a \cos\theta_a}, \tag{S4}$$

and of Snell's law,

$$\boldsymbol{n}_a \sin\theta_a = \boldsymbol{n}_q \sin\theta_q = \boldsymbol{n}_l\theta_l, \tag{S5}$$

where $\boldsymbol{n}_q$, $\boldsymbol{n}_l$. and $\boldsymbol{n}_a$ are, respectively, the complex refractive indices of the quartz window, latex and air. θ_a and θ_l are, respectively, the angle of incidence and transmission of the emitted beam relative to the quart, and θ_q is the angle at which the beam propagates through the quartz. E'_{sample} and $E'_{reference}$, respectively represent the the electric field detected when a latex sample is placed atop of the quartz window, and the electric field detected with no sample placed atop of the quartz window.

After measuring M, we can make use of the equations S4 and S5 to extract the sample's complex refractive index, n_s from the known properties of the system,

$$\begin{aligned}
\boldsymbol{n}_l \cos\theta_l &= \frac{\boldsymbol{n}_a\boldsymbol{n}_q \cos\theta_a \cos\theta_q(M+1) - \boldsymbol{n}_q^2 \cos\theta_q(M-1)}{\boldsymbol{n}_1 \cos\theta_q(M+1) - \boldsymbol{n}_a \cos\theta_a(M-1)} = X \\
\boldsymbol{n}_l &= \sqrt{X^2 + \boldsymbol{n}_q^2 \sin^2\theta_q}, \\
n_s &= Re(\boldsymbol{n}_l)
\end{aligned} \tag{S6}$$

where n_s is the real refractive index and X is merely used for simplification.
